# Sequential Vaccination Against *Streptococcus pneumoniae* Appears as Immunologically Safe in Clinically Stable Kidney Transplant Recipients

**DOI:** 10.3390/vaccines12111244

**Published:** 2024-10-31

**Authors:** Monika Lindemann, Lukas van de Sand, Nils Mülling, Kim L. Völk, Ulrich W. Aufderhorst, Benjamin Wilde, Peter A. Horn, Andreas Kribben, Adalbert Krawczyk, Oliver Witzke, Falko M. Heinemann

**Affiliations:** 1 Institute for Transfusion Medicine, University Hospital Essen, University of Duisburg-Essen, 45147 Essen, Germany; kimlouisa.voelk@uk-essen.de (K.L.V.); peter.horn@uk-essen.de (P.A.H.); falko.heinemann@uk-essen.de (F.M.H.); 2 Department of Infectious Diseases, West German Centre of Infectious Diseases, University Hospital Essen, University Essen-Duisburg, 45147 Essen, Germany; lukas.vandesand@uk-essen.de (L.v.d.S.); adalbert.krawczyk@uk-essen.de (A.K.); oliver.witzke@uk-essen.de (O.W.); 3 Department of Nephrology, University Hospital Essen, University of Duisburg-Essen, 45147 Essen, Germany; nils.muelling@uk-essen.de (N.M.); benjamin.wilde@uk-essen.de (B.W.); andreas.kribben@uk-essen.de (A.K.); 4 Division of Pediatric Surgery, Department of General, Abdominal and Transplant Surgery, University Hospital Essen, University of Duisburg-Essen, 45147 Essen, Germany; 5 Institute for Virology, University Hospital Essen, University of Duisburg-Essen, 45147 Essen, Germany

**Keywords:** *Streptococcus pneumoniae*, conjugate and polysaccharide vaccines, sequential vaccination, kidney transplant patients, alloresponse, HLA antibodies

## Abstract

**Background:** Vaccination against *Streptococcus pneumoniae* is advised for transplant recipients to reduce morbidity and mortality associated with invasive pneumococcal disease. However, data on alloantibodies after sequential vaccination (with a pneumococcal conjugate vaccine followed by a polysaccharide vaccine) are still lacking. **Methods:** In the current study, we determined HLA class I and II and major histocompatibility class I-related chain A (MICA) antibodies in 41 clinically stable kidney transplant recipients. These antibodies were measured prior to and post sequential pneumococcal vaccination over a period of 12 months. Alloantibodies were measured by Luminex bead-based assays, and pneumococcal IgG antibodies were measured by ELISA. **Results:** Over a 12-month period, the sequential analysis revealed no significant change in alloantibodies. One patient developed de novo donor-specific antibodies (DSA) 1.5 months after the first vaccination, with mean fluorescence intensities of up to 2300. These DSA became undetectable in the follow-up, and the patient showed no signs of allograft rejection. Another patient experienced a biopsy-proven borderline rejection 7 months after the first vaccination but did not develop de novo DSA. Both maintained stable kidney function. As expected, the pneumococcal antibodies increased significantly after vaccination (*p* < 0.0001). **Conclusions:** Given the overall risk of alloimmune responses in transplant recipients, we would not attribute the two noticeable patient courses to vaccination. Thus, we consider sequential vaccination immunologically safe.

## 1. Introduction

*Streptococcus pneumoniae* (*S. pneumoniae*) is a Gram-positive bacterium that forms pairs (diplococcus) and frequently colonizes the human nasopharynx [1]. Outside this region, it can cause otitis media, sinusitis, lobar pneumonia, or meningitis. It is the leading cause of community-acquired pneumonia globally [2]. In addition to local infection, *S. pneumoniae* can lead to invasive pneumococcal diseases (IPD), which carry an approximately 10% fatality rate [1,3,4]. Data by the Centers for Disease Control and Prevention (CDC) indicate that the rate of IPD in organ transplant recipients is 25 times higher than in the general population [3,4]. To lower the risk of IPD, immunocompromised individuals should be vaccinated against *S. pneumoniae* [5,6].

*S. pneumoniae* contains an estimated 500 proteins on its surface [7]. The exterior of the bacterium is covered by a polysaccharide capsule and serotypes are determined by the structure of the capsule [7]. The capsular polysaccharides are considered as the main virulence factors of *S. pneumoniae* [8]. Antibodies against these polysaccharides protect against invasive infection [8]. The capsular polysaccharides are nearly always negatively charged and thereby avoid the entrapment of pneumococci in the nasal mucus [9]. They help the bacteria to survive desiccation for many days. Because the polysaccharides cover deeper bacterial surface structures, they inhibit the binding of immunoglobulins, complement components, and C-reactive protein. As described in detail in a recent Nature Review [9], *S. pneumoniae* expresses a plethora of factors that mediate immune evasion. Key mechanisms and structures that enable *S. pneumoniae* colonization also comprise several adhesion molecules, the blockade of IgA1 and the interaction with the complement system, mucus degradation, the binding of ions necessary for immune function, the impairment of neutrophil activity, and the pro-inflammatory effects of concomitant viral infections and toxins such as pneumolysin. The current vaccines against pneumococci target capsular polysaccharides.

The sequential administration of a pneumococcal conjugate vaccine followed by a polysaccharide vaccine was recommended by the CDC and the German advisory board for vaccination (Ständige Impfkommission, STIKO) for immunocompromised individuals such as transplant recipients [5,6,10]. Conjugate vaccines, which are covalently conjugated to carrier proteins, act T cell dependently and promote B cell differentiation into memory B cells or antibody-producing plasma cells. In conjugate vaccines, the capsular polysaccharides are conjugated with the highly immunogenic cross-reactive material 197 (CRM197), a non-toxic diphtheria toxoid protein. CRM197-specific type 2 helper T cells (Th2) interact with B cells that have bound and internalized the polysaccharide-CRM197 complex via polysaccharide-specific IgM, and subsequently present the processed CRM197 protein together with HLA class II to effector T cells [11]. This type of adaptive immune response is characterized by a change in antibody isotypes. On the contrary, polysaccharide vaccines contain a group of T-cell-independent, polyvalent antigens, that is, they can activate B cells without T cell help by crosslinking numerous B cell receptor molecules [12]. There are currently few clinical studies that demonstrate an increase in pneumococcal antibodies after sequential pneumococcal vaccination in solid organ transplant recipients [13,14].

The question of whether immune activation after vaccination may induce allograft rejection has been a matter of debate for several years [15,16,17,18,19,20]. As demonstrated by Locke et al. [12], proinflammatory conditions, including infection, minor surgical procedures, traumatic injury, and major medical events (e.g., myocardial infarction) may increase alloantibodies. However, data on alloresponses after sequential pneumococcal vaccination are still lacking in transplant recipients.

The objective of the present study was to sensitively monitor (subclinical) alloresponses prior to and post sequential vaccination against pneumococci in patients who have undergone kidney transplantation. The vaccination regimen comprised a 13-valent conjugated vaccine (PCV13), followed by a 23-valent polysaccharide vaccine (PPSV23). The highly sensitive Luminex™-technology-based bead assay was used to measure antibodies against human leukocyte antigen (HLA) class I and class II and against major histocompatibility class I-related chain A (MICA). In parallel, we evaluated pneumococcal IgG antibodies, monitored the occurrence of antibodies against donor-specific antigens (DSA), and assessed the incidence of allograft rejection. We also monitored kidney function. Finally, we tested whether alloantibodies, pneumococcal antibodies, or kidney function differed between females and males.

## 2. Materials and Methods

### 2.1. Patients

**Figure 1:**
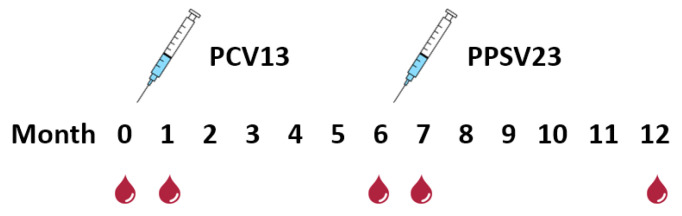
Study design. Sequential pneumococcal vaccination and blood collection in 41 kidney transplant recipients. The blood droplets indicate the time points of blood collection. The first vaccination was performed with a 13-valent conjugated vaccine (PCV13), the second with a 23-valent polysaccharide vaccine (PPSV23). Month 0 indicates baseline (pre-vaccination) and months 1–12 follow-up after the first vaccination. For example, month 7 means 7 months after the first vaccination and 1 month after the second vaccination.

A total of 41 clinically stable kidney transplant recipients were included in this cross-sectional, single-center study (Table 1). The median age of the cohort was 59 years (range 33–77 years), with 10 female and 31 male participants. The patients were vaccinated in a sequential manner against *S. pneumoniae*, with an interval of 6 months between both doses. The median interval between the (last) kidney transplantation and the first vaccination was 38 months (ranging from 3 months to 338 months, i.e., 28 years). In order to be included in the study, participants had to meet the following criteria: stable allograft function (defined as a change in serum creatinine concentration below 15% within one month prior to vaccination), an interval of at least three months since kidney transplantation, and the absence of clinical infection, allograft rejection, and pregnancy. Patients who had previously been vaccinated against pneumococci were excluded from the study. The patients were only vaccinated against pneumococci and did not receive a combined vaccination. However, vaccinations against other pathogens were not explicitly excluded during the study period of 12 months. Overall, the patients had a similar level of immune suppression. Blood samples were collected immediately prior to the two vaccinations, one month after the first, and one and six months after the second vaccination (months 7 and 12 after the first vaccination) (Figure 1). Thus, we sequentially tested five blood samples per patient. This study was approved by the institutional review board of the University Hospital Essen (14-5858-BO), and written informed consent was obtained from all participants. It was conducted in accordance with the Declarations of Helsinki and Istanbul and its subsequent amendments.

### 2.2. Vaccines and Vaccination

The first pneumococcal vaccination was performed with 0.5 mL of the 13-valent conjugate vaccine Prevenar 13^®^ (PCV13, Pfizer, Berlin, Germany) that consists of the serotypes 1, 3, 4, 5, 6A, 6B, 7F, 9V, 14, 18C, 19F, 19A, and 23F. Each of the serotypes is contained at a concentration of 2.2 µg/dose, with the exception of serotype 6B, which is present at a concentration of 4.4 µg per dose. This vaccine is an aluminum-adsorbed conjugate vaccine, where the polysaccharides are individually conjugated to a nontoxic mutant form of diphtheria toxin (CRM197). As additives, it contains 5 mM succinate buffer containing 0.85% NaCl and 0.02% polysorbate 80, at pH 5.8, as well as aluminum phosphate at a dose of 0.125 mg [21].

The 23-valent polysaccharide vaccine Pneumovax 23^®^ (PPSV23, MSD Sharp & Dohme, Haar, Germany), an inactivated vaccine, was used six months later for the second vaccination. The vaccine is unconjugated and contains the pneumococcal serotypes 1, 2, 3, 4, 5, 6B, 7F, 8, 9N, 9V, 10A, 11A, 12F, 14, 15B, 17F, 18C, 19F, 19A, 20, 22F, 23F, and 33F. The vaccine is formulated in phenol and contains <1 mmol sodium chloride per dose (0.5 mL) [22]. Both vaccines were injected into the deltoid muscle.

### 2.3. Determination of HLA and MICA Antibodies

All patient samples were tested for IgG antibodies against HLA class I and II and MICA using Luminex™-technology (LABScreen-Mixed Beads, catalog ID LSM12NC26_024_01, One Lambda/Thermo Fisher, Canoga Park, CA, USA) in accordance with the manufacturer’s instructions and as previously described in detail [23,24,25,26,27]. If results for HLA class I or II antibodies were positive, the Luminex™ Single Antigen Beads (SAB) assay, provided by the same manufacturer, was also employed to ascertain whether the HLA antibodies were donor-specific (DSA) (catalog IDs LS1A04NC26_014_00 (LABScreen Single Antigen Beads for HLA class I) and LS2A01NC26_016_01 (LABScreen Single Antigen Beads for HLA class II)) [24,25,26,27].

The Luminex™ assays facilitate the identification of antibodies against antigens that are coupled to fluorescently labeled polystyrene microbeads. As compared to the classical ELISA technique, the Luminex™ method is a multiplex assay that can analyze multiple analytes simultaneously in a single reaction, e.g., antibodies against different HLA molecules. These antibodies bind to the surface of color-coded microbeads using a blend of different fluorescent intensities of two dyes. The commercially available Luminex HLA antibody detection assays provide a higher throughput of samples, the capability of automation, and higher sensitivity as compared to the ELISA. The LABScreen-Mixed Beads Assay comprises twelve beads specific for HLA class I antibodies, five for HLA class II, and two for MICA, respectively. Reactions against each bead were scored as 1 (negative), 4 (undetermined), or 8 (positive). Individual reactions for HLA class I, HLA class II, or MICA were then summed up; yielding antibody score values of 12–96, 5–40, or 2–16, respectively, as described previously [23,27]. We performed two evaluations with two different cutoffs: A normalized background ratio above 3.0 or above 4.5 was defined as positive, and a range between 2.0 and 3.0 or 3.0 and 4.5, respectively, was defined as undetermined. Finally, a ratio below 2.0 or 3.0 was defined as negative. The first cutoff was used in previous studies of our group [23,27], and the cutoff of 4.5 is the currently applied cutoff for routine samples in our laboratory. Moreover, we evaluated the cumulative mean fluorescence intensities (MFI), where we also summed up responses for antibodies against HLA class I, HLA class II, and MICA, respectively.

### 2.4. Determination of Antibodies Against Pneumococci

The presence of antibodies against *S. pneumoniae* was determined by an ELISA which detects IgG antibodies against 23 pneumococcal serotypes (VaccZyme™, The Binding Site, Schwetzingen, Germany). The assay was conducted in accordance with the instructions provided by the manufacturer.

### 2.5. Statistical Analysis

Data were analyzed using GraphPad Prism 8.4.2.679 (GraphPad Software, San Diego, CA, USA) and IBM SPSS Statistics version 25 (Armonk, New York, NY, USA) software. The time course of antibodies was analyzed by 1-way ANOVA, considering a series of five paired samples per patient. Correlation between numerical variables was analyzed by Spearman test. Data in females and males were compared by Mann–Whitney *U* test. Two-sided *p*-values < 0.05 were considered significant.

## 3. Results

### 3.1. Kinetics of Antibody Responses

**Figure 2:**
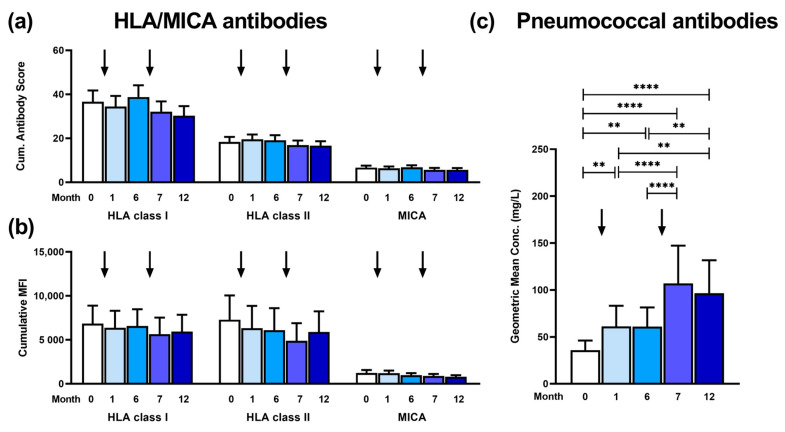
Time-course of antibodies determined in 41 kidney transplant recipients prior to vaccination (month 0, M0) and after vaccination against pneumococci (months 1 to 12, M1–M12). The first vaccination was performed at month 0 (directly after blood collection) with a 13-valent pneumococcal conjugate vaccine and the second vaccination at month 6 with a 23-valent pneumococcal polysaccharide vaccine. The time points of vaccination are indicated by arrows. The left panels show antibodies against human leukocyte antigen (HLA) class I and II and major histocompatibility class I-related chain A (MICA), which were expressed either as cumulative (cum.) antibody scores as detailed in the Methods section (**a**) or as cumulative mean fluorescence intensity (MFI) (**b**). Panel (**c**) indicates IgG antibodies against *S. pneumoniae*. Antibodies against HLA and MICA are presented as mean and standard error of the mean (SEM), pneumococcal antibodies as geometric mean and 95% confidence interval. To compare the results at the various time points, we used a 1-way ANOVA, the Friedman test with Dunn’s multiple comparisons test (** *p* < 0.01; **** *p* < 0.0001).

In 41 kidney transplant recipients, antibodies against HLA class I and II and MICA were determined sequentially, from month 0 (prior to vaccination) until month 12 post-vaccination (Figure 2a). Neither after the first vaccination with the PCV13 (follow-up at month 1) nor after the second vaccination with PPSV23 (at month 7) did we observe a change in HLA or MICA antibodies. In detail, the cumulative antibody score (mean values at months 0, 1, 6, 7, and 12) remained at a similar level for HLA class I (37, 34, 39, 32, and 30), HLA class II (18, 20, 19, 17, and 17) and MICA (7, 6, 7, 6, and 6). The mean cumulative MFI value showed a similar pattern, and no increase was observed after vaccination (Figure 2b).

In contrast, IgG antibodies against pneumococci showed a 1.9-fold increase after the first vaccination (*p* < 0.01 at month 1) and a 3.2-fold increase after the second vaccination (*p* < 0.0001 at month 7), as compared to month 0 (Figure 2c). The antibody concentration increased from a geometric mean of 36 mg/L at month 0 to 61 mg/L at month 1 and month 6 and then further increased to 107 and 97 mg/L at months 7 and 12, respectively. 

### 3.2. Patterns of Antibodies Prior to and Post Vaccination

**Figure 3:**
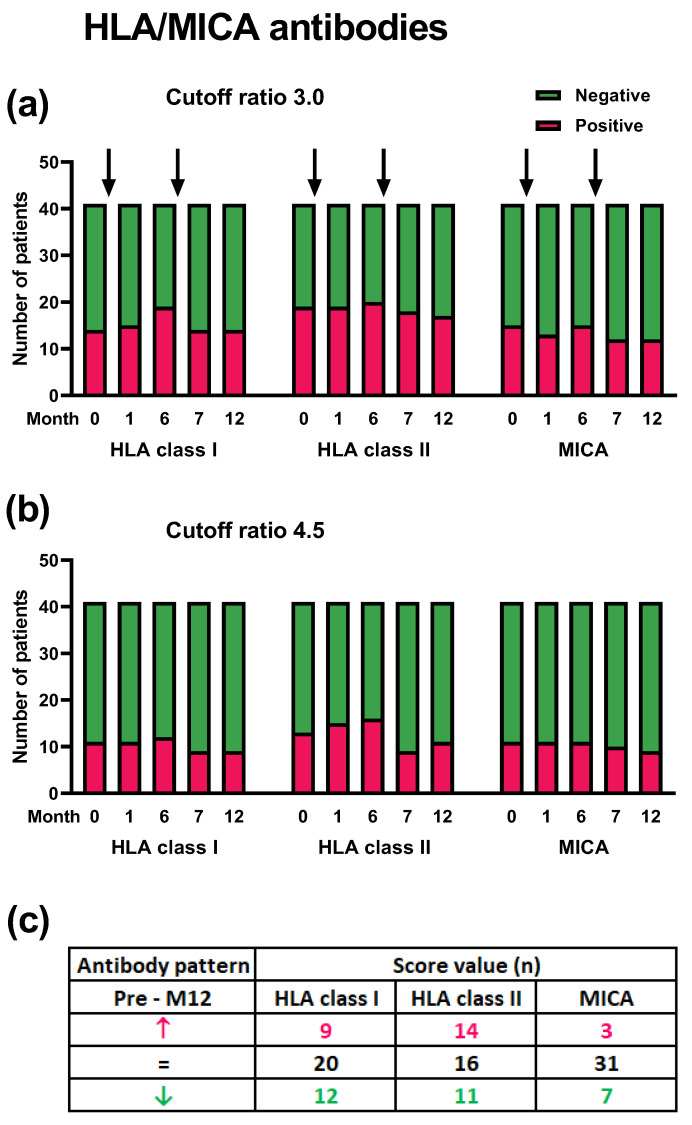
Patterns of antibodies in 41 kidney transplant recipients prior to vaccination (month 0, M0) and after vaccination against pneumococci (months 1 to 12, M1–M12). The time points of vaccination are indicated by arrows. Panel (**a**,**b**) indicate the number of patients with positive or negative antibody responses against human leukocyte antigen (HLA) class I and II and major histocompatibility class I-related chain A (MICA), setting the cutoff for positive responses at a ratio of 3.0 (**a**) or 4.5 (**b**), respectively. Panel (**c**) shows changes in the cumulative antibody score, determined pre-vaccination (month 0) and at month 12, i.e., after two vaccinations. Red symbols/numbers indicate patients with an increase (the score in month 12 was at least 1 higher than in month 0), black with constant values, and green with a decrease.

Apart from considering the mean antibody responses, we classified antibodies against HLA and MICA as positive or negative, or we followed up the individual courses of 41 kidney transplant recipients (Figure 3). In summary, there was no significant difference in antibody responses between the five time points. The number of patients with positive antibody responses for HLA class I, HLA class II, or MICA did not increase after the first or second vaccinations. This was observed irrespective of the cutoff ratio set to define a positive response, which was either 3.0 or 4.5 (Figure 3a,b). When comparing data prior to vaccination and at month 12, the number of patients with an increase and decrease in the cumulative antibody score was similar. In detail, 9 patients showed an increase, 20 showed constant values, and 12 showed a decrease in HLA class I antibodies. The respective numbers were 14, 16, and 11 for HLA class II or 3, 31, and 7 for MICA antibodies (Figure 3c). In this analysis, an increase was defined as a difference of ≥1 (month 12–month 0).

### 3.3. Correlation of HLA and MICA Antibodies with Clinical Outcome and with Patient Characteristics

One out of 41 patients, a 60-year-old female, developed de novo DSA 1.5 months after the first vaccination (anti-HLA-A1: 1600 MFI; anti-HLA-B60: 2300 MFI) but no biopsy-proven rejection within the follow-up period (Table 2, ID1). Thirteen months after the first vaccination, these low MFI values decreased (anti-HLA-A1: 800 MFI; anti-HLA-B60: 2000 MFI), and 25 months after this vaccination, the MFI values further declined (anti-HLA-A1: 550 MFI; anti-HLA-B60: 1000 MFI). This female patient was vaccinated 10 months after her first kidney transplantation and already displayed very high levels of cumulative antibody scores prior to vaccination (HLA class I: 96, HLA class II: 32), which even decreased at some time points after vaccination. The kidney function remained constant throughout the study period (estimated glomerular filtration rate (eGFR) of 61–69 mL/min/1.73 m^2^ and serum creatinine concentration of 1.1–1.2 mg/dL)).

In a second patient, a 56-year-old female, a biopsy-proven borderline rejection was detected 7 months after the first vaccination (ID2). However, she did not develop de novo DSA, and her cumulative antibody scores remained at a similar level. The patient already displayed DSA prior to vaccination, which even showed a slightly lower MFI value at month 1 after vaccination (anti-HLA-B44 at months 0 and 1:2200 MFI and 1300 MFI, respectively). At month 16 after vaccination, DSA were no longer detectable. The patient was vaccinated 22 months after her first kidney transplantation. After vaccination, no change in kidney function could be observed. Even at the time of the borderline rejection, the allograft function remained constant (eGFR of 38 mL/min/1.73 m^2^ and serum creatinine concentration of 1.6 mg/dL).

In eight further patients (ID3–8), who also received their first allografts, we observed a major increase in the cumulative antibody score for HLA (at least 10) between month 0 and month 12 (three for HLA class I, four for HLA class II, and one for both, marked red in Table 2). However, none of those patients showed DSA or allograft rejection. Three patients constantly displayed HLA class I and II antibodies (but no DSA) prior to and post vaccination (ID3–5), whereas five patients always had negative results for HLA antibodies, as defined by Luminex™ assays (ID6–10).

In these ten patients (ID1–10) with DSA or borderline rejection or a major increase in the cumulative antibody score, mean kidney function did not change significantly over time, as analyzed by time-series analyses (1-way-ANOVA). The mean eGFR values at months 0, 1, 6, 7, and 12 were 55, 51, 56, 52, and 52 mL/min/1.73 m^2^. In the remaining 31 patients, the respective eGFR values also remained at a constant level (46, 47, 47, 46, and 45 mL/min/1.73 m^2^).

**Figure 4:**
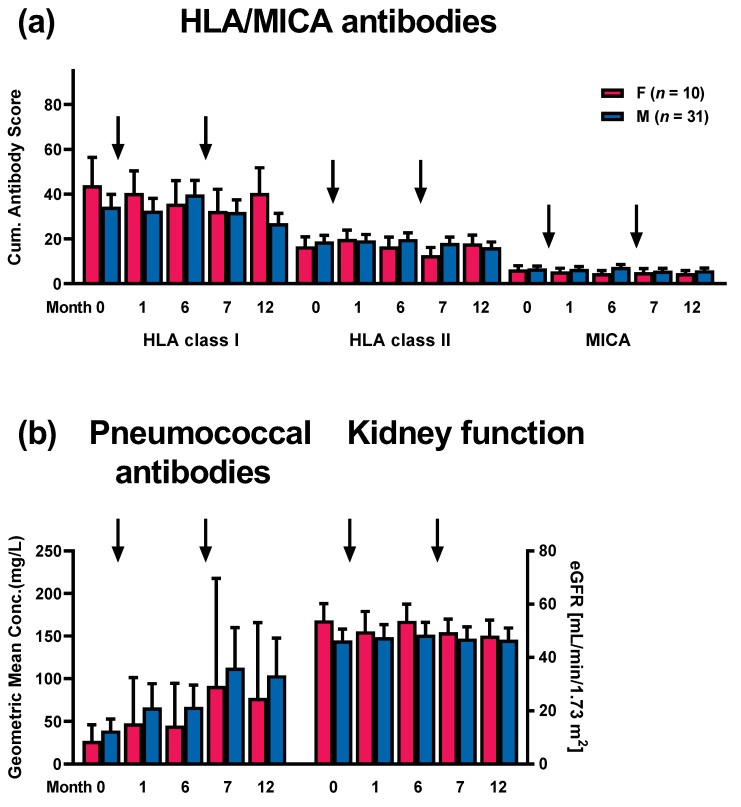
Comparison of antibody patterns in female and male kidney transplant recipients prior to vaccination (month 0, M0) and after vaccination against pneumococci (months 1 to 12, M1–M12). The time points of vaccination are indicated by arrows. Panel (**a**) indicates cumulative antibody scores for antibodies against human leukocyte antigen (HLA) class I and II and major histocompatibility class I-related chain A (MICA); panel (**b**) indicates IgG antibodies against pneumococci and kidney function (estimated glomerular filtration rate, eGFR). Data on alloantibodies and kidney function are presented as mean and standard error of the mean (SEM), and data on pneumococcal antibodies as geometric mean and 95% confidence interval.

As females may be sensitized against HLA due to pregnancy, we addressed the question of whether HLA or MICA antibodies either prior to or post vaccination were dependent on patient sex (Figure 4a). However, females and males did not differ significantly at any time point in their cumulative antibody score, as determined by the Mann–Whitney *U* test. However, HLA class I antibodies were slightly higher in females at months 0, 1, and 12. IgG antibodies against pneumococci and kidney function (eGFR) also did not differ significantly between both groups (Figure 4b). But males tended to show higher pneumococcal antibody concentrations at all time points, whereas the eGFR was slightly higher in females.

Finally, Spearman analysis yielded no significant correlation between HLA or MICA antibodies and age, the interval between transplantation, and vaccination or kidney function (eGFR).

Taken together, the data indicate that sequential vaccination with PCV13 and PPSV23 did not lead to an increase in HLA or MICA antibodies. Even in those patients with clinically noticeable courses or a major increase in cumulative antibody scores, kidney function remained stable overall. Moreover, we could not find significant differences in the course of antibodies or in kidney function between females and males.

## 4. Discussion

Taken together, our data argue against the stimulation of alloresponses after sequential pneumococcal vaccination. This result is consistent with a previous publication of our group where kidney transplant recipients received a single vaccination with PPSV23 [23]. Since the vaccine used in the current vaccination regimen, the conjugate vaccine PCV13, induces T-cell-dependent immunity, it could have been possible that the stimulation of T cells leads to cross-reactivity, which may result in alloresponses against the kidney allograft, i.e., the formation of donor-specific antibodies or finally to allograft rejection and the deterioration of kidney function. However, the occurrence of DSA with low MFI values in one patient (which further declined in the follow-up) and the occurrence of one borderline rejection without any change in kidney function may be even less adverse courses than expected within a cohort of 41 patients and a 12-month follow-up. According to registry data, the annual rate of de novo DSA formation after kidney transplantation is 2.5–5% [20,28,29], and that of rejection episodes is 3.6% to 6.4% [20,30]. Moreover, a similar number of patients showed an increase and a decrease in cumulative antibody scores for HLA and MICA within the 12-month follow-up, also arguing against the potential harm of the sequential pneumococcal vaccination.

In 2022, a 20-valent PCV vaccine (PCV20, e.g., Prevenar 20^®^, Pfizer, Berlin, Germany) was authorized in the European Union [31]. Thereafter, its use was evaluated by the German advisory board for vaccination (STIKO), which in 2024 decided to recommend it for the vaccination of adults with immunodeficiency [32]. Like Prevenar 13^®^, Prevenar 20^®^ is conjugated to the CRM197 carrier protein, it contains 2.2 µg of each serotype per dose, except for serotype 6B (4.4 µg/dose); it contains succinate and polysorbate 80, and it is adsorbed to aluminum phosphate at 0.125 mg/dose [33]. Due to its composition, it can be assumed that our data on alloimmunity after vaccination with Prevenar 13^®^ can be transferred to Prevenar 20^®^.

A recent report on 63 kidney transplant recipients, similar to the current study, indicated that two doses of a SARS-CoV-2 mRNA vaccine did not induce anti-HLA antibodies or a significant deterioration of the eGFR [34]. In a further kidney transplant cohort, two out of 100 patients showed de novo DSA after receiving two doses of a SARS-CoV-2 mRNA vaccine [35]. However, in none of the vaccinated patients was an episode of clinically evident acute cellular or antibody-mediated rejection observed, and the authors concluded that SARS-CoV-2 vaccination was not associated with changes in DSA. The findings of the studies on SARS-CoV-2 vaccination were in accordance with the experience with the H1N1 influenza pandemic of 2009. Early reports after the influenza pandemic suggested an alloreactivity to HLA from the H1N1 vaccine (and potent adjuvants have been postulated as causative), but the subsequent review of a large cohort of vaccinated solid organ transplant recipients failed to observe an association [20,27,36]. The review by Mulley et al. [20]—which is the first systematic review to assess de novo DSA and rejection episodes after vaccination in solid organ transplant recipients—included eight prospective controlled clinical studies and did not show an increased rejection risk with vaccination compared with no vaccination (RR 1.12, 95% CI 0.75 to 1.70).

Of note, our own previous data in female kidney transplant recipients indicated that there may be a sex-dependency of HLA antibody formation after a single vaccination with PCV13, and we discussed that females may be more susceptible to the induction of (non-specific) HLA antibodies after vaccination [27]. While 29 males tended to show even lower HLA class I and II antibodies after pneumococcal vaccination, HLA antibodies increased significantly at months 1 and 12 after vaccination in 18 females (*p* < 0.05) [27]. In the current study, we overall observed higher cumulative antibody scores for HLA class I in females than in males. However, this trend could not be observed for HLA class II or for MICA. In line with our previous observation, McCune et al. [35] described that the presence of DSA before SARS-CoV-2 vaccination was associated with subsequently increased MFI values or with new DSA after vaccination (*p* = 0.001). A similar phenomenon could occur in females after pneumococcal vaccination if they were pre-sensitized due to pregnancies. However, a larger data set is mandatory to definitely address this point.

Why could infection or vaccination induce alloresponses? Memory T cells that are cross-reactive to herpes viruses, especially cytomegalovirus or Epstein–Barr virus, and alloantigens [37,38] as well as inflammation and cytokine production could trigger/enhance alloresponses [39]. Cytomegalovirus infection has been identified as a risk factor for acute and chronic renal allograft rejection [18,40,41]. Immunity to herpes viruses vs. *S. pneumoniae* or the viruses SARS-CoV-2 or influenza is obviously different. While herpes viruses persist for life, the later microorganisms are eliminated after recovery from the infection. Persisting viruses could be harmful to the allograft, whereas microorganisms that are completely eliminated usually appear to be harmless.

The frequency of HLA antibodies we observed by Luminex™ was higher than previously published when setting the cutoff at a ratio of 3.0 (34–46% for HLA class I and 41–49% for HLA class II) and similar to previous studies when setting the cutoff at a ratio of 4.5 (22–27% for HLA class I and 22–39% for HLA class II) [42,43]. Previously, two large studies reported a frequency of HLA antibodies of 30% (*n* = 1014) [42] or of 27% (*n* = 4943) [43]. In our previous study, in which kidney transplant recipients received a single vaccination with PPSV23 [23], the frequencies of HLA and MICA antibodies were overall similar to those found in the current study (using a cutoff ratio of 3.0).

Zhou et al. [44] proposed that “in addition to the adaptive immune response of T and B cells against an alloantigen, MICA is also capable of setting in motion the mechanisms of innate immunity. Co-stimulation by engagement of NK cells might have the effect of potentiating the T and B cell response”. It was hypothesized that MICA antigens play a role in human allograft rejection by activating both humoral as well as cellular mechanisms [45]. Data from the 14th International Histocompatibility and Immunogenetics Workshop confirmed this hypothesis and indicated that MICA antibodies were significantly and independently associated with reduced kidney allograft survival in deceased donor grafts, providing strong evidence for the involvement of these antibodies in graft rejection [46]. The frequency of MICA antibodies in the current study of kidney transplant recipients (22–37%, depending on the cutoff) was in the range of a recent publication by Ming et al. (25%) [47].

Immunosuppressive treatment after transplantation—to prevent allograft rejection—leads to impaired cellular and humoral immunity [48]. Corticosteroids induce this impairment of T and B cell immunity by a blockade of transcription factors such as nuclear factor-κB (NF-κB), the calcineurin inhibitors cyclosporine A and tacrolimus by inhibiting the transcription of cytokine genes, especially IL-2, and mycophenolate mofetil by the blockade of DNA synthesis. In a previous study, we showed that four weeks after vaccination, the number of serotypes against which protective pneumococcal antibody concentrations were detectable was significantly lower in 43 kidney-transplant recipients as compared to 75 vaccinated healthy controls, pointing to impaired humoral immunity in kidney transplant recipients [49]. The sum of IgG antibody concentrations (against pneumococci), however, was only non-significantly lower in transplant patients than in the controls (approximately 16% lower). We are not aware of any pneumococcus-specific T cell data analyzing the effect of immunosuppressive treatment. But after vaccination against other viruses, influenza A and B, T cell responses in kidney transplant recipients vs. healthy controls were even more severely suppressed [50]. Influenza-specific proliferative responses were 3-fold lower and those of IFN-γ secretion were 3- to 9-fold lower. In the current study, however, we did not compare a transplant patient cohort with healthy controls.

There are some limitations to this study that need to be considered, such as the lack of a control group or the nature of the study, which is a single-center study. Moreover, it has been discussed that potent adjuvants such as squalene could trigger immune responses that may not be induced after applying vaccines with less potent adjuvants such as aluminum [27]. It is therefore important to emphasize that the results of the current study relate to the vaccines we used and that the results may be different when using more potent adjuvants. Thus, our findings should be generalized with caution.

In conclusion, the current data on 41 kidney transplant recipients argue against an increase of (subclinical) alloresponses after sequential pneumococcal vaccination. Thus, a vaccination scheme containing PCV13, which induces T-cell-dependent immunity, appears to be immunologically safe in clinically stable kidney transplant recipients.

## Figures and Tables

**Table 1:** Characteristics of 41 kidney transplant recipients vaccinated sequentially against *S. pneumoniae*.

Parameter	Median (Range) or Number (No.)
Median age (range), years ^1^	59 (33–77)
Patient sex (female/male)	10/31
Median interval TX-vaccination (range), months	38 (3–338)
Mean eGFR (range), mL/min/1.73 m^2^	
Pre-vaccination	48 (17–109)
Month 1 post-vaccination	48 (15–109)
Month 6 post-vaccination	50 (17–106)
Month 7 post-vaccination	48 (15–105)
Month 12 post-vaccination	47 (16–102)
Immunosuppression, no. ^1^	
Cyclosporine A	5
Tacrolimus	30
Mycofenolate mofetil	27
mTOR inhibitors	9
Corticosteroids	38
Kidney transplantation, no.	
1st	37
2nd	4

^1^ At the time of the first blood collection; eGFR-estimated glomerular filtration rate; mTOR-mammalian target of rapamycin.

**Table 2:** Characteristics of ten kidney transplant recipients with donor-specific antibodies against human leukocyte antigens (HLA), with borderline rejection, or with a major increase of the cumulative antibody score for HLA *.

ID	Sex	Age	HLA Ab **		Cum. Ab Score HLA Class I					Cum. Ab Score HLA Class II					eGFR				
			Class I	Class II	0	1	6	7	12	0	1	6	7	12	0	1	6	7	12
1	F	60	−/+	−/−	96	96	96	92	96	32	32	32	11	26	68	61	69	69	63
2	F	53	+/+	−/−	82	74	82	85	88	11	11	22	15	19	36	38	36	38	29
3	M	62	+/+	+/+	36	53	64	24	46	40	40	40	40	40	29	28	31	31	31
4	F	57	+/+	+/+	12	24	12	12	30	11	20	5	5	20	37	NT	NT	36	31
5	M	47	+/+	+/+	96	96	96	96	96	5	26	22	26	22	106	91	106	91	96
6	M	59	−/−	−/−	12	12	92	96	45	5	5	40	40	14	72	NT	81	76	84
7	M	68	−/−	−/−	12	12	12	12	12	29	17	5	25	40	25	25	20	20	18
8	F	76	−/−	−/−	12	12	12	12	12	5	14	25	17	29	76	NT	53	69	67
9	M	50	−/−	−/−	12	21	12	12	12	21	36	15	21	36	40	54	58	43	50
10	M	59	−/−	−/−	33	32	35	45	54	5	5	8	8	15	60	59	56	47	50

* Defined as increase of at least 10 between month 0 and month 12 after vaccination (marked red). ** Human leukocyte antigen antibodies (HLA Ab) as defined by Luminex Mixed Bead/Single Antigen Bead assay prior to/after vaccination against pneumococci. NT-not tested.

## Data Availability

The data presented in this study are available on request from the corresponding author. The data are not publicly available due to privacy restrictions.
